# Effect of a brief art therapy intervention on anxiety and pain in emergency department patients: a randomized open-label trial

**DOI:** 10.1186/s12245-026-01185-2

**Published:** 2026-03-12

**Authors:** Tanguy Espejo, Ornella Galvani, Clarisse Vaz, Hélène Gerhard-Donnet, Marie-Josée Brochu-Vez, Olivier Hugli, Francois-Xavier Ageron

**Affiliations:** 1 Department of Internal Medicine, Bienne Hospital Center, Biel/Bienne, Switzerland; 2 Emergency Department, Lausanne University Hospital, Rue du Bugnon 46, Lausanne, CH-1011 Switzerland

**Keywords:** Art therapy, Pain, Anxiety, Analgesia, Emergency department

## Abstract

**Background:**

Pain and anxiety are common in emergency department (ED) patients, yet their management is often focused on pharmacological interventions. Art therapy, a non-pharmacological approach, has shown promise in alleviating psychological distress, but its effectiveness in acute care settings remains understudied.

**Methods:**

This study aimed to evaluate the impact of art therapy on pain and anxiety in ED patients presenting with acute pain. A randomized controlled trial was conducted in a single ED with patients randomized to art therapy or a control group. Participants in the intervention group engaged in a 15–20-minutes art therapy session, while the control group waited without intervention. Pain and anxiety were measured using the Visual Analog Scale (VAS) and the State-Trait Anxiety Inventory (STAI-Y) at baseline and after the intervention. Exploratory analyses examined interactions between morphine use, baseline pain, and art therapy effectiveness.

**Results:**

Of the 340 patients screened, 103 completed the study (48 in the AT group, 55 in the control group). There were no significant differences in anxiety levels between the art therapy and control groups (mean difference − 2 mm, *p* = 0.610). Pain intensity was significantly lower in the control group (*p* = 0.011). In exploratory analyses, patients treated with morphine and experiencing high baseline pain levels had a significant reduction in anxiety (VAS − 18.6 mm, *p* = 0.004).

**Conclusion:**

Art therapy did not significantly reduce anxiety or pain in the general ED population. However, it showed potential as an adjunctive therapy for patients with severe pain and anxiety, particularly those receiving morphine. Further research is needed to explore the effectiveness of art therapy in this subset of patients and its potential as part of non-pharmacological pain management strategies in acute care settings.

**Trial registration:**

The study was approved by the regional ethic committee (CERVD 2021 − 01344) and registered on https://clinicaltrials.gov (NCT04997434) on 19 July 2021.

**Supplementary Information:**

The online version contains supplementary material available at 10.1186/s12245-026-01185-2.

## Introduction

### Background

Pain is the most common reason for seeking medical care, with around 60% of ED patients reporting pain on admission. Over the past two decades, improving pain management in the ED has become an increasingly important focus [1–3]. Although opioids improve pain control, their use is associated with adverse effects and the risk of addiction, leading to a rising interest in non-pharmacological therapies such as acupuncture, music interventions and virtual reality [4–8]. The ED environment, characterized by high stress and uncertainty, often exacerbates patients’ anxiety, particularly while waiting for medical consultation and awaiting diagnosis [9, 10].

Although the appropriate treatment of pain has been investigated frequently, anxiety management in the ED remains relatively underexplored [11, 12]. The ED environment is characterised by high patient turnover, constant noise, frequent interruptions, and prolonged waiting periods, all of which can intensify anxiety and pain [11–13]. Unlike inpatient wards, EDs pose additional challenges to the implementation of non-pharmacological interventions such as art therapy (AT), including constraints of time, space, and staff availability. Paradoxically, these stressors may increase the need for supportive interventions aimed at alleviating distress [14]. Investigating the feasibility and potential benefits of AT in this specific environment is therefore particularly pertinent. Anxiety can amplify the perception of pain, negatively influencing patients’ overall experience and satisfaction with care [13, 14]. The World Health Organization has highlighted the significant role of the arts in enhancing health and well-being [15]. AT leverages artistic expression, combining creative process and the practice of making art to promote health and well-being across various areas, including mental health, pain management and rehabilitation [16–18]. Modern AT differs from classic AT by emphasizing the process of artistic creation to promote mental health and well-being [19]. Unlike traditional art psychotherapy, which may involve analysing the artwork, modern AT focuses on the therapeutic benefits of the creative process itself. Modern AT uses all artistic techniques such as drawing, origami, music and writing. AT can also serve to refocus attention, which may decrease pain and anxiety by redirecting focus toward non-painful stimuli. This creates a competition between the AT experience and the pain signal for the brain’s limited attentional resources allocated to nociception [20, 21].

Low-grade evidence suggests that AT may yield positive outcomes across various patient groups, in somatic, psychological, and behavioural health. Reported benefits include reductions in physical and emotional distress during breast cancer treatment, lower rates of depression in long-term haemodialysis patients and improvement in chronic pain in cancer patients [22, 23]. A new meta-analysis concluded that AT had the potential to improve various patient outcomes, and predominantly mental health outcomes [24]. Bedside AT was shown to reduce pain and anxiety during hospitalization [25], suggesting that it may be effective in the ED. Indeed, AT was shown to reduce pain and anxiety of ED adolescents admitted with a painful condition [26].

### Importance and goals of this investigation

While numerous studies have explored the benefits of AT in patients with chronic illnesses, its use in acute care settings, such as EDs, remains limited [27] and to our knowledge, no randomized trials have assessed the impact of AT on both acute pain and anxiety in this context. In the ED, where patients are often overwhelmed by the rapid and impersonal workflow, AT may offer a means of helping individuals regain a sense of control and emotional balance [25]. Therefore, the aim of this study was to evaluate the effectiveness of AT in reducing anxiety and pain during waiting time among patients presenting with acute pain in an ED setting.

## Methods

### Study design and setting

This was an open label randomized controlled trial conducted in a single ED, with a yearly census of 45,000 consultations. The study took place in the ED of the Lausanne University Hospital, a 1,500-bed University hospital in Lausanne, Switzerland, that serves as the primary care centre for the city of Lausanne and a tertiary care centre for the region and neighbouring states. The study was performed from August 2021 to November 2021. The study was approved by the regional ethic committee (CER-VD 2021 − 01344) and registered on https://clinicaltrials.gov (NCT04997434) on 19 July 2021. The study was in accordance with the Consolidated Standards of Reporting Trials guidelines (CONSORT; details in the Supplemental Table 1) [28].

### Participants

All patients aged 18 years or older presenting to the ED with acute pain (Visual Analog Scale (VAS) of 30 millimetres (mm) or more) and whose conditions required a wait for diagnostic tests or interventions were assessed for eligibility. Baseline pain and anxiety assessments were performed upon arrival in the ED examination room and prior to randomization. Patients may have received routine analgesic treatment before inclusion, as per standard ED care. Pre-inclusion analgesic use was recorded. Patients presented with a range of common ED pain conditions, including chest pain, trauma-related conditions, abdominal pain and gastrointestinal disorders, headaches and neurologic disorders, and other acute pain presentations (see Table 1 for detailed distribution). The majority of the patients were awaiting blood test results or radiological investigations rather than procedural interventions. Exclusion criteria included clinically instability (defined as an immediate life-threatening condition requiring urgent medical intervention), inability to understand French or to use a VAS, inability to provide consent due to cognitive impairment or intoxication, incarceration, or prior enrolment in the study. Patients meeting all inclusion criteria and no exclusion criteria were enrolled by a member of the research team during regular working hours.

Patients or members of the public were not involved in the design, conduct, reporting, or dissemination plans of this study. Participant feedback was limited to post-intervention assessments.

### Intervention

#### Definition of art therapy

AT is a psychotherapeutic approach that uses artistic creation as a medium to promote health and well-being. Sessions are always conducted by a trained art therapist working within a medical or institutional framework, in collaboration with the multidisciplinary team. The focus is on the patient’s preserved resources and potential, rather than solely on symptoms or pathology.

In French-speaking Europe, the term “modern AT” is used to distinguish this approach, which emphasises the therapeutic benefits of the creative process itself, without systematic analysis of the artwork, from more traditional forms of art psychotherapy. For clarity and consistency with international standards, this manuscript uses the term “AT” to refer to this practice.

The therapeutic process may involve various art modalities, such as drawing, origami, or writing, selected according to patient preferences and feasibility in the ED setting. Rather than serving as diversion, these activities aim to facilitate sensory, emotional, and cognitive engagement within a clinician-guided therapeutic process, supporting emotional regulation and adaptive coping in the acute care setting.

#### Study setting and intervention procedure

The study took place in an ED examination room. Before the intervention, patients received limited information about the study aims to minimize bias, as blinding was not possible. Patients were informed that the study aimed to investigate the effect of an AT session. Because the intervention was minimal risk and conducted during routine ED waiting time, participants first provided oral consent after receiving this general information. To prevent patients from focusing on their pain or anxiety, forming expectations, or otherwise biasing the study, they were not informed that the research aimed to assess anxiety and pain while waiting in the ED with or without AT. After providing oral consent, patients were randomized in 1:1 ratio between the intervention and a control group. Patients were randomized using a block size of five in the RedCap^®^ randomization module, hosted at Lausanne University Hospital [29]. Following completion of the 30-minute assessment, participants received full information about the study objectives and provided written informed consent. Participants could decline written consent at that stage, in which case their data were excluded from analysis, in accordance with ethics committee approval.

Modern AT is a paramedical profession with an educational level equivalent to a master’s degree. Art therapists of this study (O.G. and C.V.) were trained in France and obtained a professional license to practice from the University of Tours. In the intervention group, certified art therapists began each session with a brief structured clinical interview (5 to 10 min). This interview explored not only the participant’s interests but also their current emotional state, presenting concerns related to the ED visit (e.g., fear, tension, uncertainty), physical limitations, and capacity for engagement. Based on this rapid therapeutic assessment, the therapist identified one or more clinically appropriate modalities and therapeutic directions, which were then discussed collaboratively with the patient. The final choice was therefore made by the patient within a therapeutically guided framework, taking into account their level of distress, need for containment or stimulation, and the constraints of the emergency setting. Available modalities included origami, drawing with coloured pencil, modelling clay, and painting contemplation (when manual activities were not feasible, e.g., due to IV lines or injuries). Although different artistic modalities were available, the duration and overall therapeutic structure of the sessions were standardized. Each session included a 5–10-minute structured clinical interview followed by approximately 15–20 min of therapist-guided art-making (a duration compatible with ED patient management, while still allowing meaningful patient engagement). The therapist remained present throughout the session. All materials used was disinfected with antiviral wipes between patients.

Patients randomized to the control group received standard of care, which consisted of waiting in the ED examination room for the results of diagnostic tests without any additional intervention. Pain and anxiety were assessed after 30 min. After data collection, the patients were given a full information regarding the study and offered to sign the full informed consent form. They could refuse to sign the form, and if so, were excluded from the analyses. No patient refused to sign the information form.

### Measurements

Pain and anxiety were assessed by VAS for all patients on arrival in the examination room prior to randomization, then approximately 30 min after baseline evaluation in both groups. In the intervention group, this corresponded to the end of the 15–20-minute art therapy session, while in the control group outcomes were measured after a 30-minute waiting period. Minor variations related to ED workflow may have occurred but no systematic timing difference between groups was intended. The assessment of anxiety was completed using a standardized questionnaire. The STAI-Y (State-Trait Anxiety Inventory) is a questionnaire for assessing state anxiety (in the present moment) and trait anxiety (over time) [30]. In this study, we used only the state anxiety section.

An evaluation grid enabled the art therapists to assess anxiety and pain during the session, as well as the patient’s engagement in the therapeutic art-making process. This grid was used as a clinical observation tool to document aspects of patient engagement such as participation in the creative process, attentional focus, emotional expression, and interaction with the therapist. The grid was completed by the art therapists delivering the intervention and served exclusively for clinical monitoring during the session. It was not included in the statistical analysis. The evaluation grid is provided in the Supplementary Materials (Supplemental Table 1). All primary and secondary outcomes were based on patient self-reported measures collected at identical time points in both study groups. Blood pressure, heart rate, oxygen saturation and respiratory rate at the start and end of the procedure were recorded if available.

### Outcomes

We hypothesized that AT could reduce anxiety and pain during ED waiting time. The primary outcome was anxiety assessed by VAS (0 to 100 mm). The secondary outcomes were pain assessed by VAS and anxiety assessed by the STAI-Y. The STAI-Y (state subscale) was included as a secondary outcome to provide a validated multidimensional assessment of state anxiety and to complement the VAS measurement.

### Statistical analysis

At the time of study design, ED-specific reference data for VAS anxiety were limited. We therefore pragmatically assumed a mean VAS anxiety score of 50 mm in the control group with a standard deviation of 30 mm, based on available population-level data [31]. The clinically relevant difference for the VAS anxiety score is not well-established. In the absence of validated thresholds, we predefined a 13 mm difference as clinically relevant by analogy with published minimal clinically important difference estimates for VAS pain [32]. A sample size of 73 patients per group was required to achieve 80% power with a two-sided p-value of less than 0.05. Analyses were conducted on an intention-to-treat basis. Depending on the distribution of the data, continuous variables were compared using either Student’s t-test or the Mann–Whitney U test, while categorical variables were compared using the Chi-squared test or Fisher’s exact test. In the event of baseline imbalance in VAS anxiety, we performed an analysis of covariance (ANCOVA) to adjust for baseline characteristics requiring fewer patients than a Student’s t-test to achieve statistical power. This approach provides an unbiased estimate of the between-group effect while accounting for initial differences at baseline.

Additionally, exploratory analyses not pre-specified in the statistical analysis plan were conducted. We searched for interactions between the effect of AT and baseline VAS for pain, analgesic administration, and opioid use. The interaction models were visualized using linear regression plots according to the VAS for pain.

In addition, we present sex-disaggregated results recommended by the SAGER guidelines [33].

## Results

### Flow chart

**Fig. 1:**
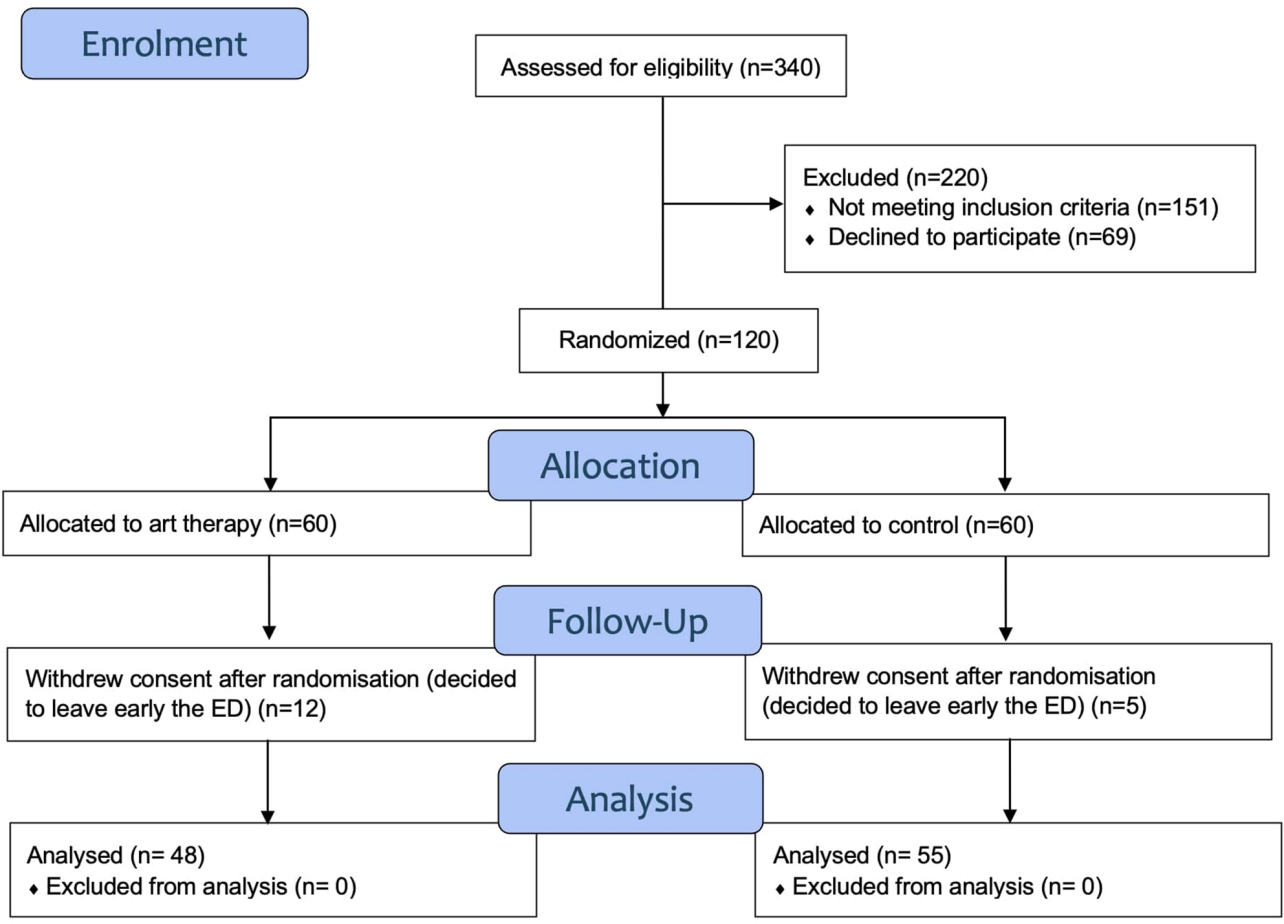
CONSORT Flow chart of the study population. Legend: The chart displays the recruitment procedure of adult ED patients with acute pain and who required waiting

During the study period, 340 consecutive patients presenting with acute pain were screened for eligibility during the research team’s working hours. Of these, 151 patients did not meet the full eligibility criteria, either because they failed to meet all inclusion criteria or met one or more exclusion criteria. A further 69 patients declined participation. This resulted in 120 patients being randomized into the two study groups. Seventeen patients withdrew their consent after randomization. A total of 103 patients were included in the study, with 48 patients in the AT group and 55 patients in the control group (Fig. 1). The study was completed within the initially planned inclusion period, which corresponded to the availability of the art therapists for the study. There were no premature interruptions. However, the initially planned number of participants was not reached, primarily due to patients withdrawing consent after deciding to leave the ED early.

### Patient characteristics

Details of demographic and clinical characteristics of the patients are reported in Table 1. The median age was 48 years (IQR 18), with no significant difference between the two groups. The proportion of female patients was 48% overall and slightly higher in the AT group. Analgesia use before inclusion was comparable between groups and included mainly acetaminophen, nonsteroidal anti-inflammatory drugs, weak opioids, and strong opioids. The types of analgesia used included mainly acetaminophen, nonsteroidal anti-inflammatory drugs (NSAIDs), weak opioids, and strong opioids. Both the baseline anxiety and pain levels were higher in the AT group. Mean STAI-Y scores were similar between groups. No adverse events or unintended effects related to the intervention were observed.

### Outcomes

**Fig. 2:**
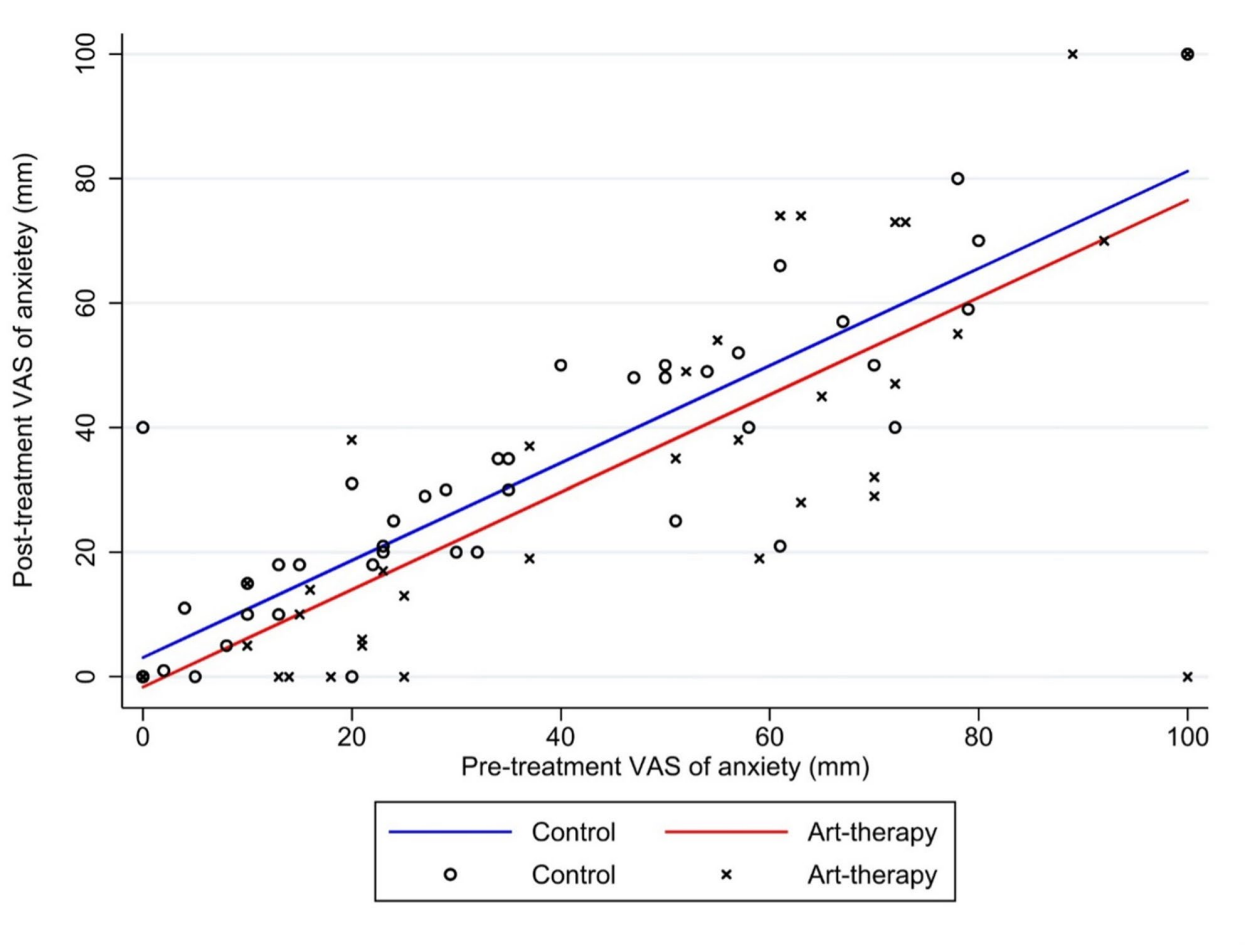
Distribution of pre- and post-intervention anxiety intensities. Legend: Analysis of covariance (ANCOVA) of the pre- and post-intervention anxiety intensities. The figure depicts the linear relationship between pre- and post-intervention anxiety for both groups. Although there is a slight separation between the regression lines suggesting a trend favouring art therapy (red line) over control (blue line), this difference was neither statistically significant (*p* = 0.101) nor clinically meaningful

The primary outcome, mean post-intervention anxiety intensity measured by VAS, was 27 mm (SD 30) in the AT group and 25 mm (SD 25) in the control group. Given baseline imbalance in anxiety levels, the primary analysis relied on a baseline-adjusted ANCOVA model. Based on this, the pre- and post-intervention VAS anxiety scores showed a general trend of reduced anxiety in the AT group. The adjusted between-group difference in post-intervention anxiety was − 4.7 mm (95% CI − 10.3 to 0.9; *p* = 0.101), This adjusted difference did not reach statistical significance and was below the prespecified threshold for clinically meaningful within-patient change (Fig. 2). Mean post-intervention pain intensity was 38 mm (SD 28) in the AT group and 25 mm (SD 23) in the control group. Because baseline pain differed between groups, we performed a baseline-adjusted analysis using ANCOVA. This model showed a non-statistically significant adjusted difference in post-intervention VAS pain between groups of 2.9 mm (95% CI − 4.3 to 10.1; *p* = 0.430).

The STAI-Y mean scores were similar between the two groups, respectively 37 (SD 10) in the AT group and 38 (SD 10) in the control group (*p* = 0.716). Regarding physiological parameters, mean systolic arterial pressure, mean heart rate, and mean oxygen saturation levels were comparable between the groups. Details are presented in Table 2.

**Table 1:** Patients’ demographic and clinical characteristics

	Art therapy (*n* = 48)	Control (*n* = 55)	*P* value
**Age**, median (IQR)	51 (18)	45 (18)	0.107
**Female sex**, n (%)	27 (56)	22 (40)	0.100
**Highest education attainment**, n (%)			0.234
None	1 (2)	1 (2)	
Mandatory school	2 (4)	8 (15)	
Secondary education, high school	19 (40)	18 (33)	
Tertiary education, university	24 (55)	14 (50)	
**Emergency Department chief complaints**, n (%)			0.393
Chest pain & cardiology	8 (17)	11 (20)	
Trauma	12 (25)	14 (25)	
Abdominal pain & gastro-intestinal disorders	14 (29)	12 (22)	
Headaches & neurologic disorders	3 (6)	4 (7)	
Others	11 (23)	14 (25)	
**Any analgesia before inclusion**, n (%)	26 (60)	28 (51)	0.333
Acetaminophen	23 (48)	25 (45)	0.803
NSAIDs	17 (35)	16 (29)	0.493
Tramadol	6 (13)	13 (24)	0.146
Morphine	9 (19)	10 (18)	0.941
**Pre-intervention systolic arterial pressure**,** [mmHg]**, mean (SD)	123 (18)	132 (21)	0.021
**Pre-intervention cardiac frequence**,** [bpm]**, mean (SD)	71 (14)	73 (18)	0.479
**Pre-intervention oxygen saturation**,** [%]**, mean (SD)	97 (4)	97 (2)	0.794
**Pre-intervention anxiety intensity**,** [mm]**, mean (SD)	37 (32)	27 (28)	0.108
**Pre-intervention pain intensity**,** [mm]**, mean (SD)	43 (26)	29 (24)	0.006
**Pre-intervention STAI-Y**, mean (SD)	40 (12)	40 (10)	0.771

This table presents the demographic and clinical characteristics of patients in the Art Therapy group (N=48) and the Control group (N=55). Data are shown as median (interquartile range [IQR]) for continuous variables or as numbers (percentages) for categorical variables. Statistical significance between groups was assessed using appropriate tests, with p-values reported

Abbreviations: bpm, beats per minute; IQR, interquartile range; mmHg, millimetre of mercury; NSAIDs, nonsteroidal anti-inflammatory drugs; SD, standard deviation; STAI-Y, State-Trait Anxiety Inventory

**Table 2:** Main and secondary outcomes

	Art therapy(n=48)	Control(n=55)	P value
**Primary outcome**			
Post-intervention anxiety intensity, [mm], mean (SD)	27 (30)	25 (25)	0.610
**Secondary outcomes**			
Post-intervention pain intensity, [mm], mean (SD)	38 (28)	25 (23)	0.011
Post-intervention STAI-Y, mean (SD)	37 (10)	38 (10)	0.716
Post-intervention systolic arterial pressure, [mmHg], mean (SD)	122 (17)	128 (20)	0.146
Post-intervention cardiac frequence, [bpm], mean (SD)	71 (15)	76 (20)	0.136
Post-intervention oxygen saturation, [%], mean (SD)	97 (3)	96 (5)	0.323

This table reports the primary and secondary outcomes for patients in the Art Therapy group (N=48) and the Control group (N=55). Data for the primary outcome (anxiety intensity) and secondary outcomes (pain intensity, STAI-Y scores, systolic arterial pressure, cardiac frequency, and oxygen saturation) are presented as median (interquartile range [IQR]) or mean (standard deviation [SD]) depending on the variable. Statistical comparisons between groups are expressed as p-values

Abbreviations: bpm, beats per minute; IQR, interquartile range; mmHg, millimeter of mercury; SD, standard deviation; STAI-Y, State-Trait Anxiety Inventory

Sex-disaggregated analysis did not find any difference between men and women; anxiety reduction − 4.5; 95%CI (-11.4; 2.3) for women and − 5.5; 95% CI (-14.5; 3.5) for men (P for interaction = 0.704).

### Exploratory analyses

Among the 103 included patients, 19 received morphine before inclusion (9 in the AT group and 10 in the control group). We found that morphine therapy and baseline VAS pain modified the effect of AT. We found an interaction between morphine and AT (p for interaction = 0.016). And we found a triple interaction between morphine, baseline VAS pain and AT (P for interaction = 0.002). In patients treated with morphine, AT showed a significant reduction in anxiety VAS scores of -18.6 mm (95% CI [-31.1, -6.0]; *p* = 0.004). AT lead to a significant reduction in anxiety VAS scores of at least − 32.0 mm (95% CI [-47.1, -17.0]; *p* < 0.001) for patients treated with morphine and with a baseline VAS pain of 60 mm or higher (Supplementary Fig. 1).

## Discussion

This randomized controlled trial evaluated the effects of AT on anxiety and pain among ED patients admitted with acute pain. Our primary outcome of anxiety reduction did not show statistically significant differences between the AT and control groups. A non-prespecified exploratory analysis suggested a possible benefit of AT for patients with severe pain and those treated with morphine. These findings are preliminary and require confirmation in studies specifically designed to test these hypotheses. Previous studies have demonstrated AT’ potential to alleviate distress and improve emotional well-being in various clinical settings, including oncology and chronic pain management [22, 23]. Literature on AT use in the ED remains sparse, but recent studies demonstrated its potential to decrease acute pain and anxiety [26].

Our study extends the limited evidence on AT by demonstrating its potential to reduce pain related anxiety, particularly in patients experiencing severe pain. This finding suggests a possible co-linearity between anxiety and pain: patients with higher initial pain levels, possibly associated with the need for morphine, may experience increased anxiety, which AT could help alleviating [34–36]. Interestingly, in another ED study, adolescents who had received an analgesic prior to the AT session experienced a greater decrease in anxiety immediately after AT completion [26]. Conversely, those without initial anxiety may derive less benefit, potentially biasing the results toward the null. This absence of observable effect in participants with low baseline anxiety may reflect a floor effect of the VAS measure, limiting the ability to detect further reductions in this subgroup. The STAI-Y and VAS may not accurately reflect the complex interplay between pain and anxiety [37, 38]. Additionally, patients receiving morphine might interpret its administration as an indication of a severe condition, which could heighten their anxiety.

The study also assessed the feasibility of implementing AT in a busy ED environment. No intervention-related adverse events were identified, supporting the short-term safety of AT in this ED setting. Although some patients withdrew due to prolonged waiting times, AT was successfully administered to a majority of participants, demonstrating feasibility in a busy ED setting. However, its implementation in other EDs may require additional resources, such as dedicated therapists and patient engagement strategies. Resource availability, particularly in high-income countries, supports potential implementation. However, in resource-limited settings, logistical and financial constraints could be significant barriers.

### Strengths and limitations

This study is one of the first randomised controlled trial to investigate the effects of AT on anxiety and pain in adult ED patients. Its pragmatic approach, conducted in a real-world ED setting, makes the findings more generalizable to similar settings. Additionally, comprehensive data collection, including VAS scores for anxiety and pain, along with physiological parameters such as blood pressure and heart rate, provided a holistic view of patient responses.

However, the study also had several limitations. First, the final sample size (103 patients) was smaller than initially planned, which may have limited the study ability to detect smaller, clinically significant effects. In addition, no validated minimal clinically important difference exists for VAS anxiety in the ED setting. The predefined 13 mm threshold used for sample size calculation was extrapolated from VAS pain literature and should therefore be interpreted with caution. Second, blinding was not possible due to the nature of the intervention, which could have introduced performance or detection biases. However, patients completed by themselves the VAS of pain and anxiety on an electronic tablet without the assistance of the investigators. Third, the validity of the intention-to-treat (ITT) analysis may be compromised by the unexpected withdrawal of some participants. Conducting the trial in an ED setting increases the likelihood of consent withdrawal, as participants may face time constraints or acute changes in their clinical status. Specifically, 12 participants in the AT group and 5 in the control group withdrew consent, which could introduce attrition bias if the reasons for withdrawal were related to the intervention or outcomes. However, most withdrawals were due to factors unrelated to the intervention, particularly early discharge or transfer to another ward during the intervention period. Under Swiss regulations, we were unable to include data from these participants, as no written consent was obtained. This limitation restricted our ability to adjust for missing outcomes and may affect the internal validity of our findings. Nevertheless, our transparent reporting of withdrawals and their distribution allows readers to critically assess their potential influence on the study’s conclusions.

Fourth, we did not anticipate that the baseline characteristics would be imbalanced between the two groups, with differences in initial anxiety and pain levels potentially affecting the outcomes. This baseline imbalance may have biased our results toward the null hypothesis, as patients with higher initial anxiety and pain levels may require different or longer interventions to achieve meaningful reductions. To address this, we therefore applied an ANCOVA test that adjusts for differences in baseline anxiety levels, a decision made *a posteriori*. Additionally, we conducted non-prespecified exploratory analyses to adjust for potential interaction. The greater responsiveness observed in patients with severe pain should be interpreted cautiously, as these analyses were exploratory and not prespecified, and may be influenced by residual confounding or limited statistical power. These results provide preliminary insights that may inform the design of future study. Fourth, the short duration of the AT sessions (15–20 min) might not have been sufficient to induce significant reductions in anxiety or pain, and the control group, which received no intervention, made it difficult to isolate the effects of AT from the natural passage of time. The variety of AT activities offered to participants could have also added variability in the therapeutic effects, and a standardized activity might have provided more consistent results. As most AT sessions were conducted during daytime hours and enrollment was restricted to the research team’s working hours, the potential influence of different ED atmospheres—particularly during night shifts or periods of higher crowding—was not explored, which may have introduced selection bias and limits the generalizability of the findings. Although outcome assessment was scheduled at approximately 30 min in both groups, small variations in timing inherent to emergency department workflow may have occurred and could have introduced measurement variability. Additionally, our intervention included AT and the presence of an additional healthcare provider. We are therefore unable to untangle the benefit due to AT alone, the presence of a therapist or to both. Furthermore, we included patients with heterogeneous presenting pain conditions (e.g., chest pain, trauma-related pain, abdominal pain, headache), which may differ in both pain quality and associated anxiety. The study was not powered to detect condition-specific effects, and differential responsiveness to art therapy according to pain type cannot be excluded. Lastly, the study only assessed immediate outcomes without long-term follow-up, limiting understanding of the sustained impact of AT in this setting.

## Conclusion

This randomized open-label trial is the first to investigate the use and effect of AT on anxiety and pain in an ED. A 15- to 20-minute AT session did not significantly reduce anxiety or pain in the overall study population. Post-hoc exploratory analyses indicate potential benefit of AT for patients with severe pain who are receiving morphine. These findings are preliminary and further trials are required to clarify this potential effect modifier. 

## Supplementary Information

Below is the link to the electronic supplementary material.

Supplementary Material 1

Supplementary Material 2: Supplemental Fig. 1: Interaction Between Baseline VAS Pain, Morphine Therapy, and the Effect of Art Therapy on Anxiety. Legend: Linear regression plots illustrating the interaction between baseline VAS for pain, analgesic administration, and opioid use on the effect of art therapy.

Supplementary Material 3: Supplemental Table 1: Art Therapy Engagement Observation Grid. Legend: This table presents the observation grid used by the art therapists during the intervention sessions to document patient engagement in the therapeutic art-making process. The grid includes items assessing observable aspects such as participation in the activity, attentional focus, emotional expression, and interaction with the therapist. The grid was completed by the art therapists during the session and was used solely for clinical monitoring of the intervention. It was not used as an outcome measure and was not included in the statistical analyses.

## Data Availability

The datasets used and analysed during the current study are available from the corresponding author on reasonable request.

## Abbreviations

AT: Art Therapy
ANCOVA: Analysis of Covariance
CI: Confidence Interval
ED: Emergency Department
IV: Intravenous
NSAIDs: Nonsteroidal Anti-inflammatory Drugs
SD: Standard Deviation
STAI-Y: State-Trait Anxiety Inventory (State subscale)
VAS: Visual Analog Scale
