# Supplementary figures and images for: Effect of a brief art therapy intervention on anxiety and pain in emergency department patients: a randomized open-label trial
Source: Int J Emerg Med. 2026 Mar 12;19:60. doi: 10.1186/s12245-026-01185-2 (PMC12983888; doi:10.1186/s12245-026-01185-2)

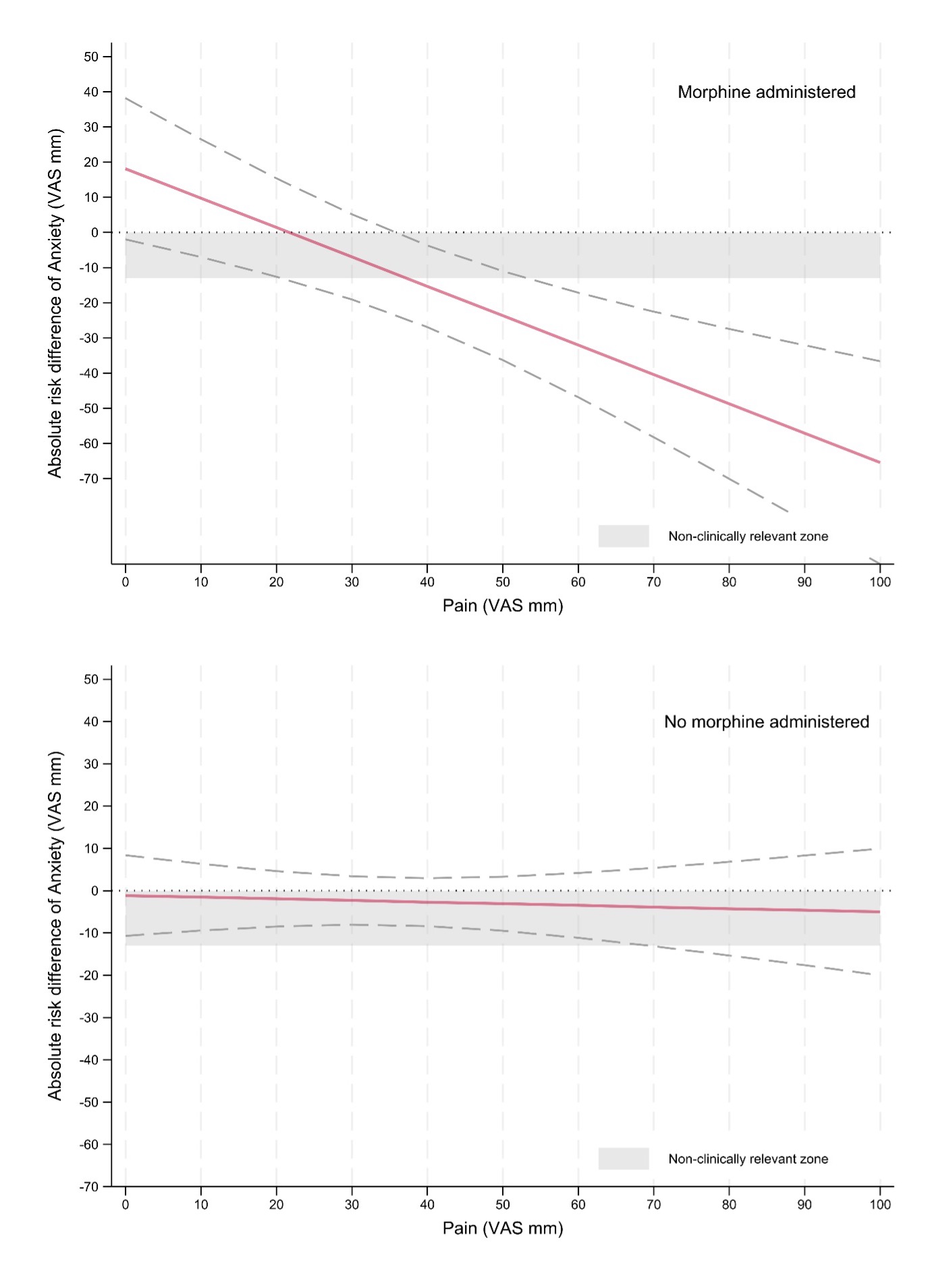

Supplement: Supplementary file 2 — Supplementary Material 2: Supplemental Fig. 1: Interaction Between Baseline VAS Pain, Morphine Therapy, and the Effect of Art Therapy on Anxiety. Legend: Linear regression plots illustrating the interaction between baseline VAS for pain, analgesic administration, and opioid use on the effect of art therapy. [file 12245_2026_1185_MOESM2_ESM.jpg]
