# Supplementary material for: Effect of a brief art therapy intervention on anxiety and pain in emergency department patients: a randomized open-label trial
Source: Int J Emerg Med. 2026 Mar 12;19:60. doi: 10.1186/s12245-026-01185-2 (PMC12983888; doi:10.1186/s12245-026-01185-2)
Supplement: Supplementary file 3 — Supplementary Material 3: Supplemental Table 1: Art Therapy Engagement Observation Grid. Legend: This table presents the observation grid used by the art therapists during the intervention sessions to document patient engagement in the therapeutic art-making process. The grid includes items assessing observable aspects such as participation in the activity, attentional focus, emotional expression, and interaction with the therapist. The grid was completed by the art therapists during the session and was used solely for clinical monitoring of the intervention. It was not used as an outcome measure and was not included in the statistical analyses. [file 12245_2026_1185_MOESM3_ESM.docx]

# Art Therapy Assessment Grid

| Observation items related to anxiety assessment | | |
| --- | --- | --- |
| **Concerns About Circumstances** | **Verbal Expression of Worry or Distress** | **Expression of Unwillingness to Undergo a Medical Procedure** |
| 5. Talks about their life  4. Talks more about their life than about the accident or being at the hospital  3. Talks equally about their life and the accident or being at the hospital  2. Talks less about their life than about the accident or being at the hospital  1. Talks only about the accident or being at the hospital | 5. None  4. Rare (1 time)  3. Regular (2-3 times)  2. Frequent (> 3 times)  1. Constant | 5. None  4. Rare (1 time)  3. Regular (2-3 times)  2. Frequent (> 3 times)  1. Constant |
|  | **Haste** | **Physical Manifestations** |
|  | \| 5. No haste \| \| --- \| \| 4. Only for non-artistic actions \| \| 3. For communication \| \| 2. For artistic action \| \| 1. For all actions \| | 5. Relaxed and serene attitude  4. Calm attitude  3. Tense and/or hesitant attitude  2. Needs to get up and move  1. Confused attitude and/or crying |
| **Physical Agitation** | **Body Position** | **Nervous Agitation, Cries** |
| 5. None  4. Rare (1 time)  3. Regular (2-3 times)  2. Frequent (> 3 times)  1. Constant | 5. Relaxed  4. Tense  3. Contractured  2. Frozen  1. Crouched | 5. None  4. Rare (1 time)  3. Regular (2-3 times)  2. Frequent (> 3 times)  1. Constant |
|  | **Tension in Hands and Feet** | **Requests Clarification on Instructions** |
|  | 5. Relaxed extremities  4. Supple extremities  3. Tense extremities  2. Contracted extremities  1. Clenched extremities | 5. None  4. Rare (1 time)  3. Regular (2-3 times)  2. Frequent (> 3 times)  1. Constant |

| **Observation items related to pain assessment** | | |
| --- | --- | --- |
| Verbal expressions of pain | Stiffens, tenses up | Presence of moaning |
| 5. None  4. Rare (1 time)  3. Regular (2-3 times)  2. Frequent (> 3 times)  1. Constant | 5. None  4. Rare (1 time)  3. Regular (2-3 times)  2. Frequent (> 3 times)  1. Constant, prevents the session | 5. None  4. Rare (1 time)  3. Regular (2-3 times)  2. Frequent (> 3 times)  1. Constant |
| Facial expressions: furrowed brow, frowned eyebrows, tightened mouth | Physical contact with the injured area |  |
| 5. None  4. Rare (1 time)  3. Regular (2-3 times)  2. Frequent (> 3 times)  1. Constant | 5. None  4. Rare (1 time)  3. Regular (2-3 times)  2. Frequent (> 3 times)  1. Constant |  |

| Observation items related to the assessment of expressed enjoyment | | | |
| --- | --- | --- | --- |
| Qualitative verbal expression of feelings | Verbalising the fact of enjoying the activity | | Facial expression of emotions (qualitative) |
| 5. Expresses positive feelings  4. Expresses moderate feelings  3. Expresses negative feelings  2. Expresses no feelings  1. Expresses feelings not related to aesthetics | 5 - Spontaneously says that he likes this activity and would like to have it at home.  4 - Says that he likes this activity.  3 - Says nothing about it.  2 - Says that he does not like this activity.  1 - Spontaneously says that he does not like this activity and would not like to have it at home. | | 5. Expresses positive emotions  4. Expresses moderate emotions  3. Expresses negative emotions  2. Expresses no emotions  1. Expresses emotions not oriented towards aesthetics |
| Quantitative verbal expression of positive feelings | Physical relaxation | | Body language expression of emotions (quantitative) |
| 5. Very often (more than 5 times)  4. Often (4 to 5 times)  3. Sometimes (2 to 3 times)  2. Rarely (once)  1. Never | 5 - Relaxed posture throughout the session  4 - Posture that relaxes gradually  3 - Posture unchanged throughout the session  2 - Posture that becomes tense gradually  1 - Closed, oppositional posture, unsuitable | | 5. Very often (more than 5 times)  4. Often (4 to 5 times)  3. Sometimes (2 to 3 times)  2. Rarely (once)  1. Never |
| Mood on Arrival | | Mood on departure | |
| 5. Enthusiastic  4. Smiling  3. Indifferent  2. Sad, worried  1. Annoyed, angry | | 5. Enthusiastic  4. Smiling  3. Indifferent  2. Sad, worried  1. Annoyed, angry | |

| Observation items related to assessing engagement in the activit | | |
| --- | --- | --- |
| Intention | | |
| Motivation | Statement of intent | Autonomy |
| 5. Wants to participate in the activity  4. Wants to participate, asks for help  3. Wants to participate after being prompted  2. Wants to participate only after insistence  1. Does not want to participate | 5. Expresses a positive intention  4. Expresses a mixed intention  3. Expresses a negative intention  2. Expresses an intention not oriented towards aesthetics  1. Expresses no intention | 5. Makes their own choices without prompting  4. Hesitates to make their own choices  3. Asks for help in choosing  2. Chooses when prompted  1. Does not make choices |
| Involvement | | |
| Adaptation of body structure | Imaginative ability | Used area |
| 5. Organises themselves physically to carry out their activity  4. Readjusts their position on their own, repeatedly  3. Adjusts their position when asked  2. Adjusts their position with assistance  1. Remains in an unsuitable position | 5. Uses imagination spontaneously and in relation to the activity  4. Uses imagination when prompted  3. Asks for help to create and imagine  2. Uses imagination unrelated to the activity  1. Does not use imagination, cannot form mental images | 5. Total (100%)  4. Partial (75%)  3. Average (50%)  2. Low (25%)  1. None (0%) |
| Implementation | Attention span | Action dynamics |
| 5. Begins the activity quickly  4. Begins the activity  3. Hesitates to begin the activity  2. Begins after being prompted  1. Does not begin the activity | 5. Continuous concentration during the activity (100%)  4. Concentration approximately 75% of the time  3. Concentration approximately 50% of the time  2. Concentration approximately 25% of the time  1. No concentration (0%) | 5. Fast pace of action  4. Steady pace of action  3. Slow pace of action  2. Jerky pace of action  1. No action |
| Engagement in artistic activity | | |
| Attitude in action | Completion of production | Taking initiative |
| 5. Spontaneously adapts attitude to carry out activity successfully  4. Readjusts attitude independently, repeatedly  3. Adapts attitude when asked to do so  2. Remains in an inappropriate attitude  1. Inappropriate attitude, does not allow activity to be carried out | 5. Successful and corresponds to its aesthetic ideal  4. Completed  3. Sloppy  2. Unfinished  1. Abandoned | 5. Often takes initiative spontaneously  4. Sometimes takes initiative spontaneously  3. Takes initiative when asked  2. Takes initiative when asked repeatedly  1. Never takes initiative |
| Expression of taste | Duration of participation | Confidence in Movement |
| 5. Exprime souvent son goût spontanément  4.Exprime quelquefois son goût spontanément  3. Exprime son goût par imitation  2. Exprime son goût sous sollicitation  1. N’exprime jamais son goût | 5. Continuous (throughout the entire session)  4. A lot (more than half of the session)  3. Very often (half of the session time)  2. A little, occasionally, sporadic  1. Never | 5. Is confident in their movements  4. Adopts confident movements if encouraged/praised  3. Adopts shy/timid movements  2. Adopts shy/timid movements only with encouragement  1. Does nothing |
|  | Signing Their Work | Naming Their Work |
|  | 5. Signs their work independently without prompting  4. Asks for help to sign  3. Signs after being prompted  2. Indifferent about signing  1. Does not wish to sign | 5. Names their work independently  4. Asks for advice to name their work  3. Names their work after being prompted  2. Indifferent about naming their work  1. Does not wish to name their work |
